# Compact Colocated Bimodal EEG/fNIRS Multi-Distance Sensor

**DOI:** 10.3390/s25175520

**Published:** 2025-09-04

**Authors:** Frédéric Hameau, Anne Planat-Chrétien, Sadok Gharbi, Robinson Prada-Mejia, Simon Thomas, Stéphane Bonnet, Angélique Rascle

**Affiliations:** University Grenoble Alpes, CEA, Leti, F-38000 Grenoble, France; anne.planat-chretien@cea.fr (A.P.-C.); sadok.gharbi@cea.fr (S.G.); robinson.pradamejia@cea.fr (R.P.-M.); simon.thomas@cea.fr (S.T.); angelique.rascle@yahoo.fr (A.R.)

**Keywords:** EEG, fNIRS, bimodal acquisition system, wearable, real environment

## Abstract

At present, it is a real challenge to measure brain signals outside of the lab with portable systems that are robust, comfortable and easy to use. We propose in this article a bimodal electroencephalography–functional near-infrared spectroscopy (EEG-fNIRS) sensor whose spatial geometry allows the robust estimation of colocated electrical and hemodynamic brain activity. The geometry allows for the correction of extra-cerebral activity (short-channel distance) as well as the computation of the spatial gradient of absorbance required in the spatially resolved spectroscopy (SRS) method. The complete system is described, detailing the technical solutions implemented to provide signals at 250 Hz for both synchronized modalities and without crosstalk. The system performances are validated during an N-Back mental workload protocol.

## 1. Introduction

For years, neuroimaging studies in the field of cognitive neuroscience have used controlled experiments where a participant is assigned a task to perform while his neural activity is recorded. Different modalities can be used, ranging from electroencephalography (EEG) to functional magnetic resonance imaging (fMRI), with specific protocols that allow the identification of relevant neuromarkers for many different brain processes such as mental workload, inhibition process, emotional response, motor activation, etc. [1]. If simplified protocols in controlled environments have improved the understanding of brain function and its functions, it remains that these paradigms are not representative of the everyday environment, made up of complex interactions, multi-factorial, evolving over time at different scales, etc.

There is now, and has been for several years, a real momentum for body-worn systems with the objective of accessing new protocols or environments more representative of real conditions. These wearable systems have, for instance, allowed the emergence of a new field of neuroscience since the 2000s: social interaction and environmental influence [2].

The most common outside-the-lab system is EEG that measures the post-synaptic response of neurons, in the microvolt range (1–10 μV), and allows quantifying the electrical activity of the brain with a temporal resolution of a few tens of ms (acquisition around 1 kHz). Because of the low volume conduction due to electrical conductivity of the skull, it is quite poorly spatially resolved. Another disadvantage is that it is quite sensitive to movement, which remains an issue for a system that should be carried out of the laboratory. That is why many studies in the field of hyperscanning are conducted using systems like functional near-infrared spectroscopy (fNIRS) less sensitive to movement [3,4]. Hyperscanning measures the brain activity of two or more participants in a simultaneous, synchronous experiment with the possibility of interacting with each other. This kind of paradigm necessarily involves portable, synchronized and robust systems to the presence of movements: hence the interest of fNIRS in this context. Such systems measure cerebral hemodynamic activity thanks to the use of near-infrared light, which is sensitive to the absorption and scattering properties of the brain tissues (scalp, skull and cortex). The neurons’ activation induces a local variation of oxygenated and deoxygenated blood concentration in the cerebral cortex, which in turn leads to a local variation of the tissue absorption. The fNIRS signal is thus a measure of the hemodynamic activity and an indirect measure of the neuron activation. The hemodynamic variations are local and have a lower temporal resolution than EEG (10 Hz).

It has been shown in previous work [5] that fNIRS provides a hemodynamic signal similar to the blood-oxygen-level dependent (BOLD) signal in fMRI with a higher temporal resolution. Furthermore, fNIRS allows estimating two types of concentrations: deoxy-hemoglobin (Hb) and oxy-hemoglobin (HbO_2_), as opposed to fMRI which only provides oxy. Due to the propagation of photons in a diffusing medium, only the superficial layers of the brain (cortex) are accessible by fNIRS, while fMRI accesses deeper structures. This makes fNIRS a preferred tool for treating prefrontal and frontal areas, which are involved in many cognitive processes [6].

EEG and fNIRS are thus two complementary modalities, both by the phenomena they measure (electrical and hemodynamic activity respectively), but also by their intrinsic spatio-temporal properties. Moreover the coupling of the two allows access to the neurovascular cerebral coupling (NVC), which is itself representative of brain activity. The coupling of EEG and fNIRS is not new, and many studies have already shown its interest. In the medical field, it has been shown that bimodality allows a better diagnosis in the context of many diseases such as Alzheimer’s [7], Parkinson’s disease [8], or depression [9]; its use can be extended to mental stress [10] but also to motor rehabilitation [11].

Moreover, the coupling of the two modalities has shown better classification results in the field of brain–computer interface (BCI) [12] or even in more conventional cognitive studies such as workload [13] or mental arithmetic [14]. It can also be used to denoise one modality using information from another modality [15].

To date, most studies use commercially independent fNIRS and EEG systems (e.g., g.tec, Artinis, NIRx, and Cortivision to cite a few wireless portable solutions) coupled on the cap and synchronized with each other usually by a soft trigger. To our knowledge, there is currently no fully integrated commercial fNIRS-EEG device. Some attempts have been made in this direction. Some companies have adopted an additional modular approach to facilitate the construction of multimodal systems. However, such systems tend to be full-head, bulky and expensive; their installation is long and tedious; the cap can even be uncomfortable in some cases. This type of system is therefore not well suited to the outdoor objective.

An alternative is to develop an integrated dedicated EEG/fNIRS system [16]. Efficient crosstalk suppression between modalities and the rejection of (large) interferences either electrical (line noise) or optical (ambient light) are the main issues to be addressed in building such hybrid instruments. Comfort and ease of use are also key components. In [17], the authors propose a bimodal EEG-fNIRS system located at the forehead developed with two EMG/EEG-channels and one dual-wavelength fNIRS channel to monitor anesthesia depth. fNIRS is especially relevant here, since EEG may not be altered when certain drugs are injected. NVC, assessed through the EEG beta bandpower and HbO_2_ concentration, estimated from fNIRS signals, is also shown to decrease during anesthesia. In [18], the wearable EEG issues are detailed with a focus on colocated EEG–fNIRS signals to address the time synchronization requirements for BCI purposes. More recently, Li et al. [19] describe a hybrid colocalized EEG/fNIRS integrated wearable patch for brain monitoring. The EEG/fNIRS system from Cui [20] developed in 2024 comprises 16 sources/16 detectors (112 fNIRS channels) and 16 EEG channels. EEG measurement is performed during ambient light slot in fNIRS recording (when LEDs are off).

**Figure 1:**
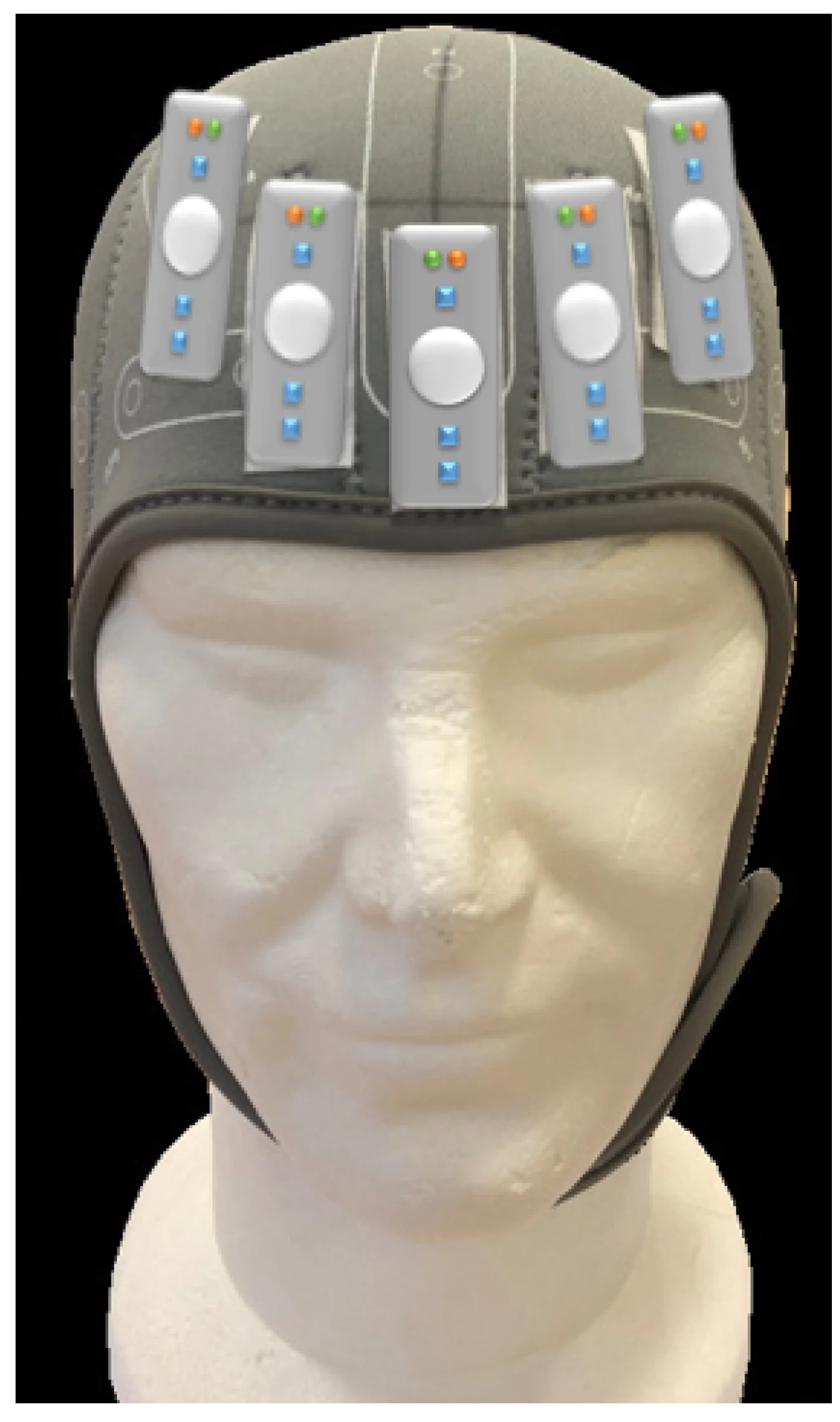
Optrode sensor node positioning area at AF8-AF7-AF4-AF3 and AFZ. (blue dots correspond to photodiodes, red and green dots correspond to LED and the white dots are the EEG electrodes).

In this paper, we propose a new concept of a colocated EEG/FNIRS node, which is called the Optrode node (contraction of optical and electrode). The main design criteria that have driven our implementation are the conformability of the sensor, its versatile and easy-to-place nature, and the colocation of electrical and hemodynamic activity measurement combined with a multi-distance geometry to improve fNIRS processing. Those criteria are clear advantages with respect to previous works. summarizes. Several nodes can be assembled in the same experimental setup to address one or more areas of interest. Each node is designed to optimize the measurement of the cerebral hemodynamic signal on the one hand and the EEG signal on the other hand. Indeed, unlike the other systems mentioned, our Optrode sensor nodeproposes a particular fNIRS geometry composed of two sources (two wavelengths) and three photodiodes (one short distance and two long distances). This innovative geometry must allow the brain signal to be corrected by the contribution of the extra-cerebral compartment on the one hand and to dissociate the systemic response from the cerebral response when present [21,22]. Moreover, the multi-distance measurement enables both the modified Beer–Lambert [23] and spatially resolved spectroscopy (SRS) method [24] in one device. The sensor has been designed for the forehead measurement area (hairless area). This restriction choice is justified for a future easy-to-use helmet integration perspective. Figure 1 shows the sensor positioning used for workload classification studies. Those studies are not detailed in this paper, which focuses on the system description, and will be published in a future second paper.

Careful attention to the design of the electronic PCBs avoids any crosstalk between the two modalities and provides a specific robust EEG signal. Finally, the chronogram and system architecture provides an fNIRS signal at a higher rate than current systems. It will allow extracting the photoplethysmography signal (PPG) from the fNIRS signal acquired on the short-distance photodiode. It gives access to physiological parameters such as the hearth rate (HR) and HR variability, which can complement neurological activity.

The present paper describes in Section 2 the developed EEG/fNIRS system from the sensor to the whole electronics structure. The characterization from an electrical and optical point of view is then given in Section 3 using phantoms. Finally, in Section 4, the system is tested during a cognitive task (i.e., memory workload), and some cerebral electrical/hemodynamic activations are discussed.

## 2. System Description

### 2.1. Optrode Sensor Node

**Figure 2:**
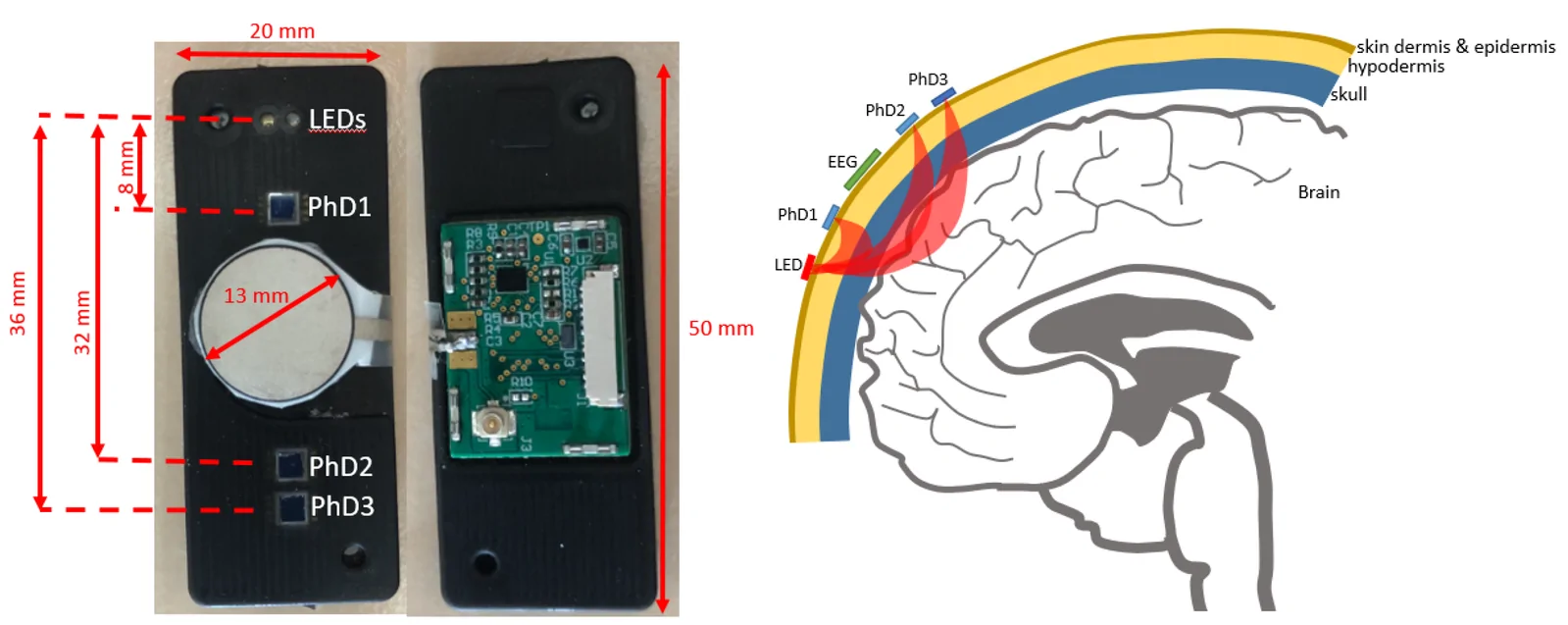
(**left**) Optrode sensor node. The electrode center is at equal distance from the LED and PhD3. Left part is in the contact with the skin. Right part is the backside of the sensor. (**right**) Simplified representation of the light transmission between the source and the detectors using a banana shape to illustrate the paths of the photos in probability.

Our system aims to study cerebral activity based on EEG/fNIRS bimodality. Thus, a monolithic sensor including the EEG and the fNIRS modalities has been developed. Such a sensor node, denoted the Optrode sensor node, is illustrated in Figure 2. The overall dimension is 50 mm × 20 mm. It includes an EEG flat disk-shaped electrode and a dual-wavelength fNIRS component with three sensing distances. The sensor has been moulded in a biocompatible black silicon and provides soft and comfortable usage.

The fNIRS part of the sensor is composed of two light-emitting diodes (LEDs), OIS-330 IT850 from OSA Opto Light, Berlin, Germany and IE740 from EPIGAP OSA Photonics GmbH, Berlin, Germany, with wavelengths $\lambda_{1}$ = 740 nm (red), $\lambda_{2}$ = 850 nm (infrared) and three photodiodes (PhDs), S16008-33 from Hamamatsu Photonics, Hamamatsu, Japan. The photodiodes are used to provide three separate source–detector distances. The photodiode PhD1 is located $d_{1}=8$ mm apart from the LEDs, and this short-distance source–detector arrangement is intended to correct the fNIRS signal from the unspecific superficial layer attenuation [25].

The two long-distance photodiodes ($d_{2}$ = 32 mm, $d_{3}$ = 36 mm) are associated with a much higher penetration depth to reach cortical tissues. One can combine the short- and long-distance photodiodes to apply the modified Beer–Lambert method to estimate the cerebral oxygenation [23]. Moreover, our design can also directly estimate the spatial gradient of the optical density by applying the spatially resolved spectroscopy (SRS) method [24].

On the backside of the fNIRS sensor, an analog front-end (AFE) has been integrated. This AFE is responsible for the LEDs activation and photodiode signal digitization. The AFE4420 is an ultra-small multisensor optical biosensing AFE from Texas Instruments, Dallas, TX, USA and supports signal acquisition of up to 16 phases with a flexible allocation of LED–PhD pairs in each phase. It offers an adjustable LED drive from 50 to 200 mA with an 8-bit programmable current source. It supports four time-multiplexed photodiode inputs with a 10 kΩ to 2 MΩ transimpedance amplifier gain. It also includes a digital subtraction at the ADC output to suppress ambient light and an automatic ambient cancellation mode in order to increase the receiver dynamic range.

The electrode on the Optrode sensor node is based on a stacked Ag/C/AgCl. This passive electrode requires the additional use of conductive gel to improve the skin–electrode contact impedance. This is acceptable, since the Optrode nodes are mostly intended for forehead use (no hair).

**Figure 3:**
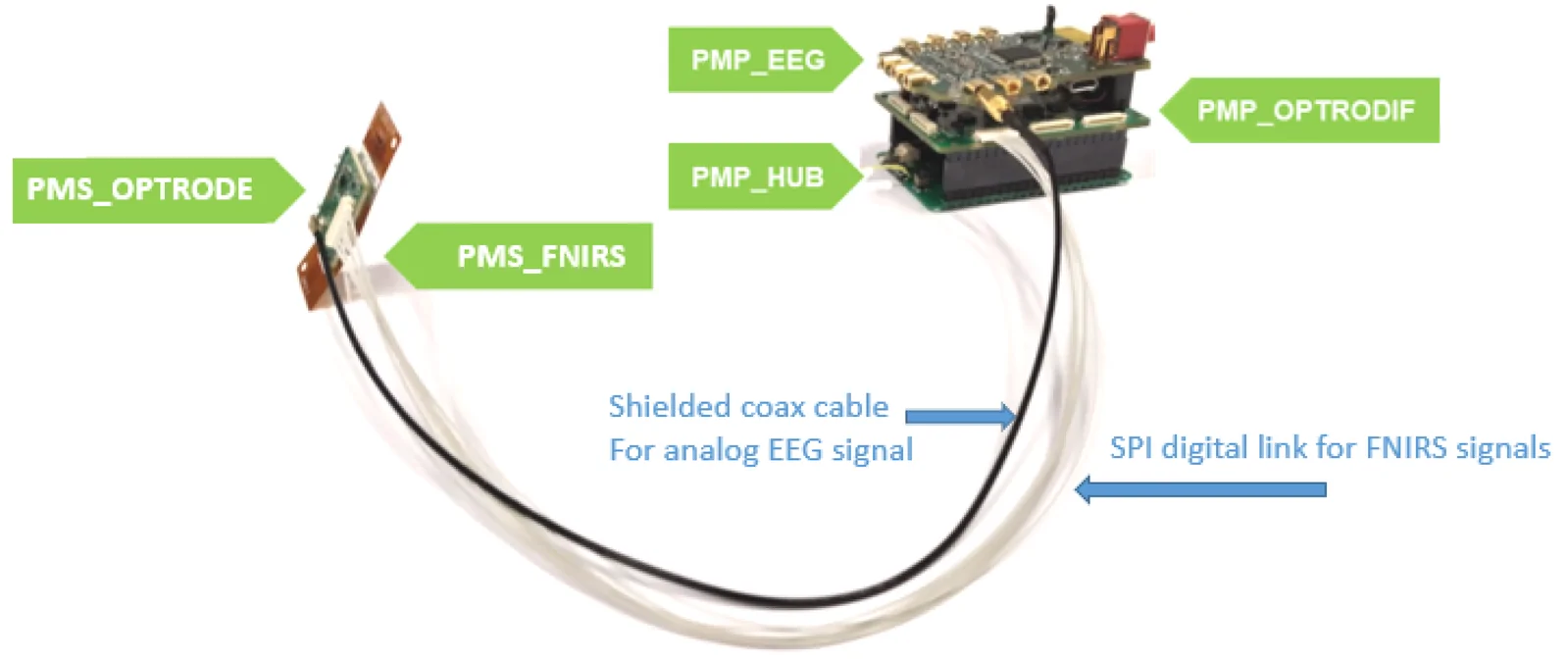
Optrode system view with one Optrode sensor node and the HubNode (dimension 80 × 70 × 65 mm).

Finally, the electrical analog signal is directly driven to a subclic connector to be fed through a shielded coaxial cable to the main EEG board where the ADS1299 AFE from Texas Instruments, Dallas, TX, USA will operate the analog-to-digital conversion. This coaxial cable solution allows minimize fNIRS and EEG signals coupling. The whole Optrode system is illustrated in Figure 3.

### 2.2. HubNode

All sensors are connected to a central unit, named HubNode, which is composed of three printed circuit boards as shown in Figure 3.

- The main board, PMP-HUB, includes the micro-controller NRF52840 from Nordic Semiconductor, Trondheim, Norway and part of the power management. This board is responsible for the finite-state machine (FSM) EEG/fNIRS measurement sequence, the Serial Peripheral Interface (SPI) setting of the different AFEs, the flash data storage and the Bluetooth pairing with the external control computer.
- The PMP-OPTRODIF board collects up to 8 fNIRS sensors digital information to concentrate the data to the PMP-HUB for storage.
- The PMP-EEG board includes the single ADS1299 AFE. It receives the analog signal of up to 8 EEG electrodes (EEG from the Optrode node or from any commercial EEG electrodes) and the necessary DRL and REF electrodes. As this board is isolated from the rest of the unit thanks to a galvanic isolation coupler (ISO7242 and ISO7341 from Texas Instruments Dallas, TX, USA), it has its own battery and power management. With its 24-bit sigma-delta analog-to-digital converters combined with a programmable gain amplifier (PGA), it offers a very high sensitivity. It can operate from 250 sps to 16 ksps. Its input-referred noise in normal mode at 250 sps is as low as 0.98 μV_pp_ peak to peak. The ADS1299 digitizes the analog signal from EEG electrodes and sends it to the PMP-HUB for storage.
- A TTL input has been added in order to enable hardware tag events for third-party systems and protocol synchronization. This tag ability is also mandatory for offline post-treatments.

### 2.3. Finite State Machine (FSM)

**Figure 4:**
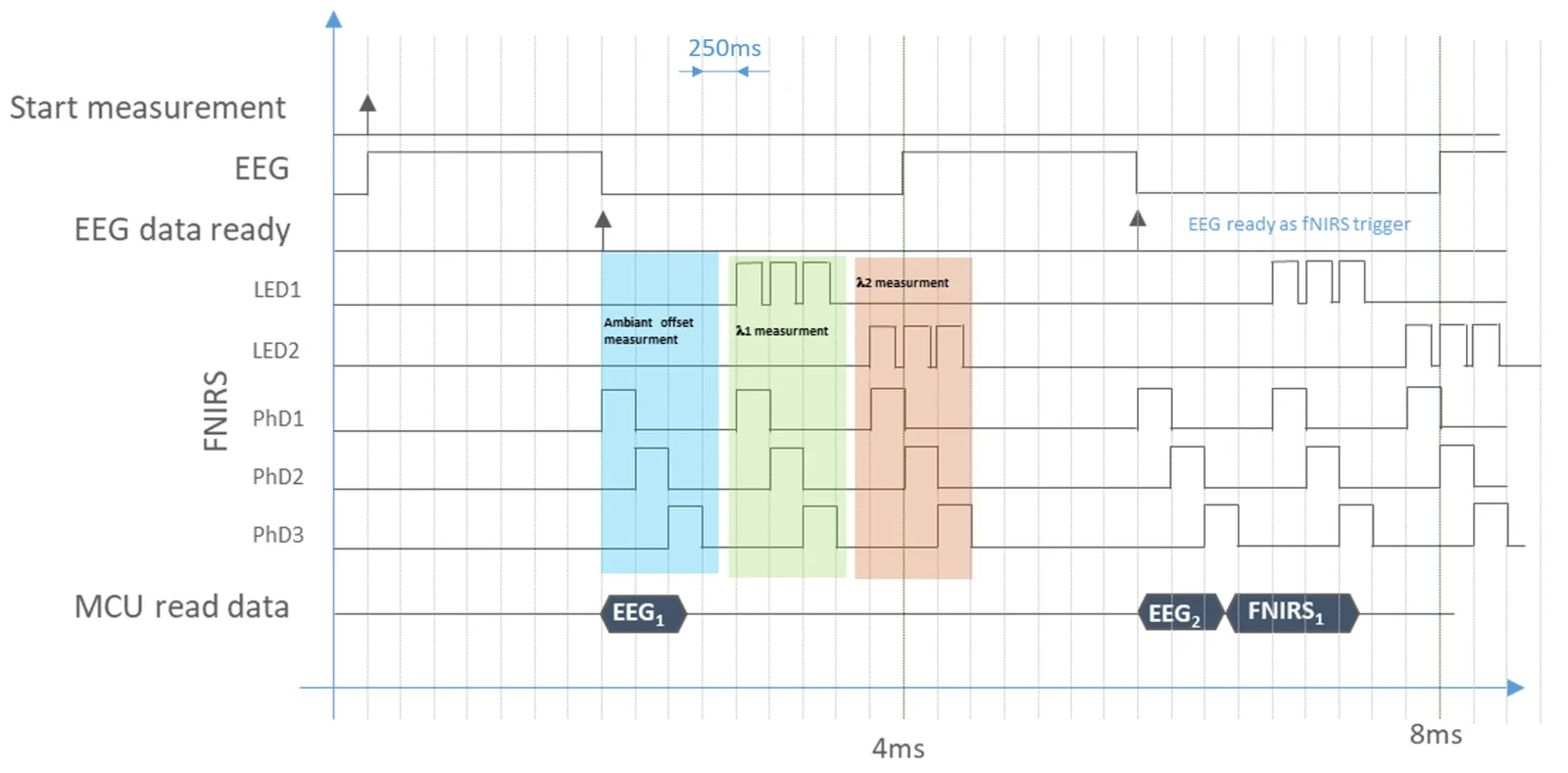
Finite State Machine chronogram with EEG and fNIRS phases (at the trigger time, ${\mathrm{EEG}}_{n}$ will be saved with ${\mathrm{fNIRS}}_{n-1}$).

In order to guarantee the synchronization between both EEG and fNIRS modalities, the system works with EEG as the master. The FSM is started by the microcontroller (MCU) with a clock frequency of 32 MHz. As shown in Figure 4, every 4 ms, a measurement is carried out. This chronogram draws some inspiration from the work in [26]. First, the FSM triggers the ADS1299 for EEG measurement. It is only at the end of the EEG data conversion, when the EEG data ready of the ADS1299 rises, that the fNIRS measurement starts. The fNIRS measurement consists of three main slots: ambient light measurement, 740 nm LED illumination and 850 nm LED illumination measurements. For each slot, the three photodiodes are sequentially measured. The number of slots is controllable, but it is mandatory that the end of the fNIRS measurement slot ends before the next EEG data are ready, since this rising will trigger the next fNIRS measurement sequence. Each time EEG data are ready, the MCU reads data from each sensor and saves them.

### 2.4. fNIRS Sensor Calibration

**Figure 5:**
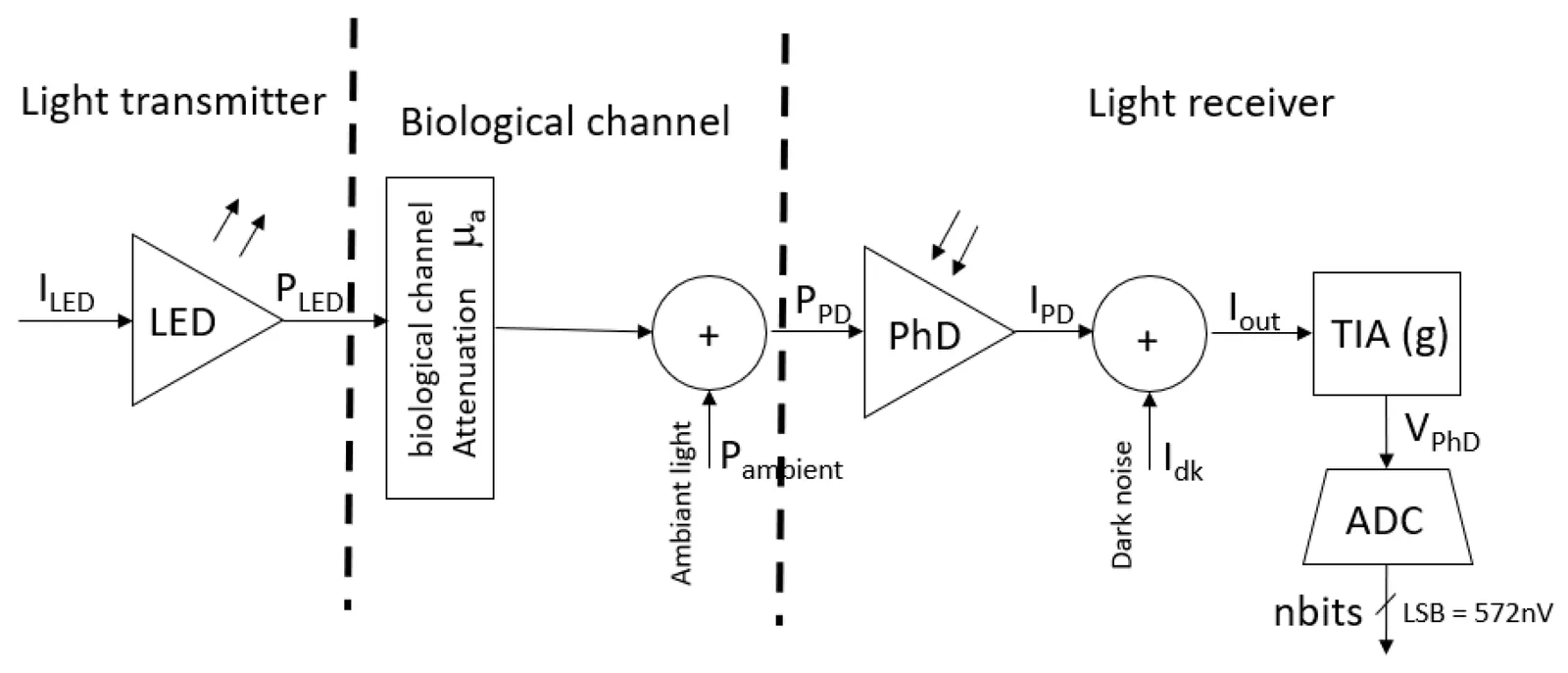
One-channel fNIRS block diagram. Double arrows represent emitted/emitted light.

The system aims to measure the biological tissue attenuation, which is an image of the oxygenation and deoxygenation of the blood. Thus, a calibration of the light source and photodiode is required. Figure 5 shows the block diagram of the fNIRS system. An LED provides a light power source ($P_{\mathrm{LED}}$, which goes through the biological tissue (extra-cerebral and cerebral tissue). The received power at the photodiode side ($P_{\mathrm{PhD}}$) is converted in current before it is amplified, converted into voltage through a transimpedance amplifier (TIA), and finally converted into a digital nBit signal. The TIA gain is set by the feedback resistor $R_{f}$ and can be programmed from 10 kΩ to 2 MΩ. The gain $g_{\mathrm{TIA}}$ between the input current and output differential voltage of the TIA is equal to $2R_{f}$. In our case, we set a gain of $R_{f}$ = 25 kΩ for PhD1 and $R_{f}$ = 2 MΩ for PhD2–PhD3.

#### 2.4.1. LED Calibration

**Figure 6:**
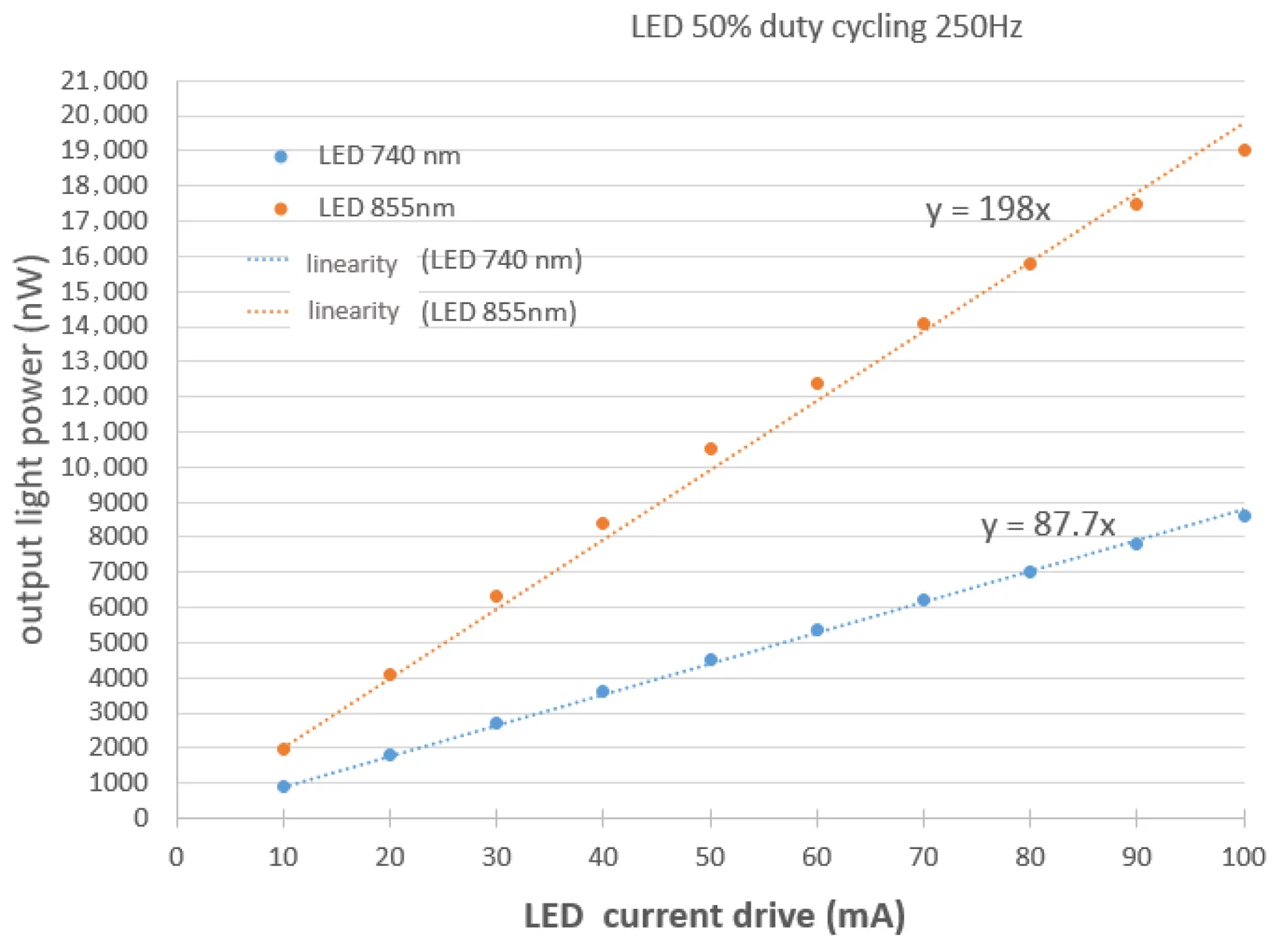
Optrode LED output power versus current drive.

A linear relationship ($P_{\mathrm{LED}}=aI_{\mathrm{LED}}$) links the output optical power $P_{\mathrm{LED}}$ directly available at the LED surface with the LED driving current $I_{\mathrm{LED}}$. The goal of LED calibration is to find the coefficient *a* that expresses the linear relationship between them. To do so, each LED is placed directly in contact with the Newport photodetector (Newport 1918 from MKS instruments, Andover, MA, USA) with a $S_{\mathrm{Newport}}=1$ cm^2^ active surface. The LED control is set at its maximum duty cycle (50% at 250 Hz). The power measurement will take into account the LED packaging molded in the black silicon. As shown in Figure 6, the two LEDs, $\lambda_{1}=740$ nm and $\lambda_{2}=850$ nm, have the expected slope given in the datasheet.

#### 2.4.2. Photodetector Calibration

In order to characterize the fNIRS sensor photodetector, a 50 cm cylindrical light chamber is used. Two calibrated Thorlabs LEDs are used in this case (one for each wavelength) so that we can control exactly the power that impinges the photodetector at the tube output. At a distance of 50 cm, we measured the relations $P_{\mathrm{Newport}}\;\left[\mathrm{nW}\right]=1.1940\cdot I_{{\mathrm{LED}}_{T}}\;\left[\mathrm{mA}\right]$ for $\lambda_{1}$ = 740 nm, $P_{\mathrm{Newport}}\;\left[\mathrm{nW}\right]=1.3058\cdot I_{{\mathrm{LED}}_{T}}\;\left[\mathrm{mA}\right]$ for $\lambda_{2}$ = 850 nm.

In the first photodetector calibration experiment, light measurements were successively achieved using the Newport1918 and the Optrode photodetector. The Thorlabs LED $I_{{\mathrm{LED}}_{T}}$ was then controlled with a current ramp. The optical power obtained with the commercial photodetector was denoted $P_{\mathrm{Newport}}$. When the Newport photodetector was replaced by the Optrode fNIRS sensor (PhD surface is 2.4 cm × 2.4 cm), the voltage $V_{PhD}$ was measured and converted back to intensity using the chosen TIA gain.

The photodiode sensitivity at a given wavelength is computed as shown below:

(1) $$s\left(\lambda ;I_{{\mathrm{LED}}_{T}}\right)=\frac{V_{PhD}/g_{TIA}}{P_{PhD}}=\frac{V_{PhD}/g_{TIA}}{\frac{S_{\mathrm{PhD}}}{S_{\mathrm{Newport}}}P_{\mathrm{Newport}}}$$

We note that $s_{i}$ is the sensitivity of the *i*-th photodiode.

**Figure 7:**
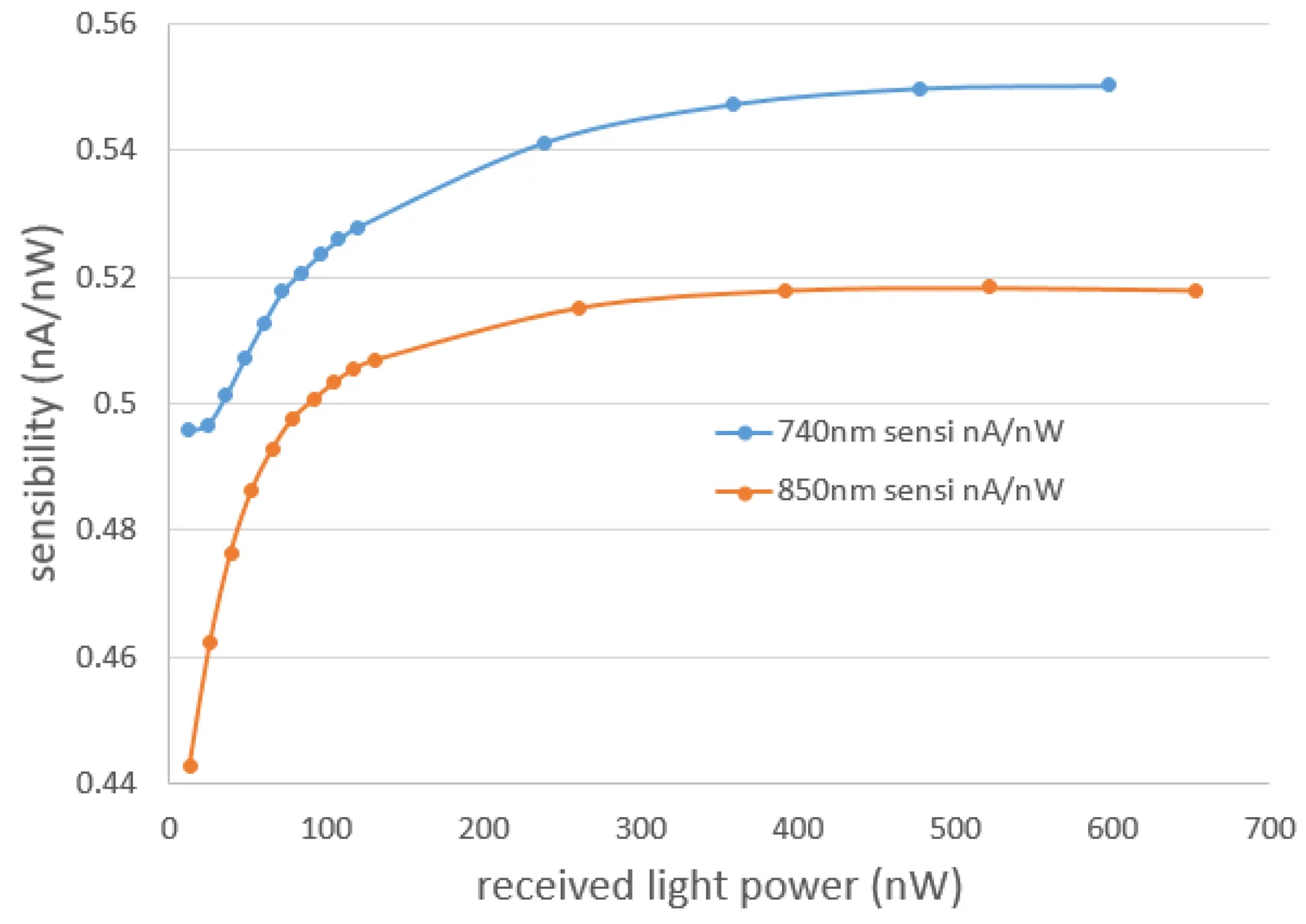
Optrode photodiode sensitivity versus received light power.

The absolute sensitivity is given in Figure 7 and is about 0.5 nA/nW, which is close to the component datasheet.

In practice, only sensitivity ratios are used, leading to the following calibration coefficient:

$$c_{ij}=\frac{s_{i}\left(\lambda_{j}\right)}{s_{1}\left(\lambda_{j}\right)}$$

These values are reported in Table 1. The values are close to unity since the photodetectors have the same characteristics. The difference observed is mainly due to the packaging (black silicone), which can be more or less adjusted to each photodiode. This calibration factor is important, since it makes it possible to take into account the components aging over time or possible cleaning defects.

In the second photodetector calibration experiment, we generate for each wavelength the same exact optical power at the output of the Newport tube. This second calibration step allows to define the following quantum yield:

$$\eta_{ij}=\frac{V_{PhD}^{i}\left(\lambda_{j}\right)}{V_{PhD}^{i}\left(\lambda_{1}\right)}$$

for each TIA gain and photodiode. The obtained quantum yields are presented in Table 2.

In fNIRS studies, each photodiode signal is first corrected by its associated ambient signal measurement (cf. chronograph) and then ‘normalized’ with respect to photodiode PhD1 and wavelength $\lambda_{1}$. This corrected signal is given below:

(2) $${\tilde{V}}_{PhD}\left(d_{i},\lambda_{j},t\right)=\frac{\eta_{ij}}{c_{i1}}V_{PhD}\left(d_{i},\lambda_{j},t\right)$$

## 3. System Characterization

In this section, the characterization of the whole system is described. First, an electrical characterization is made for each modality. The crosstalk between EEG and fNIRS modalities is then quantified.

### 3.1. Optrode Sensor Characterization

#### 3.1.1. EEG Performances

**Figure 8:**
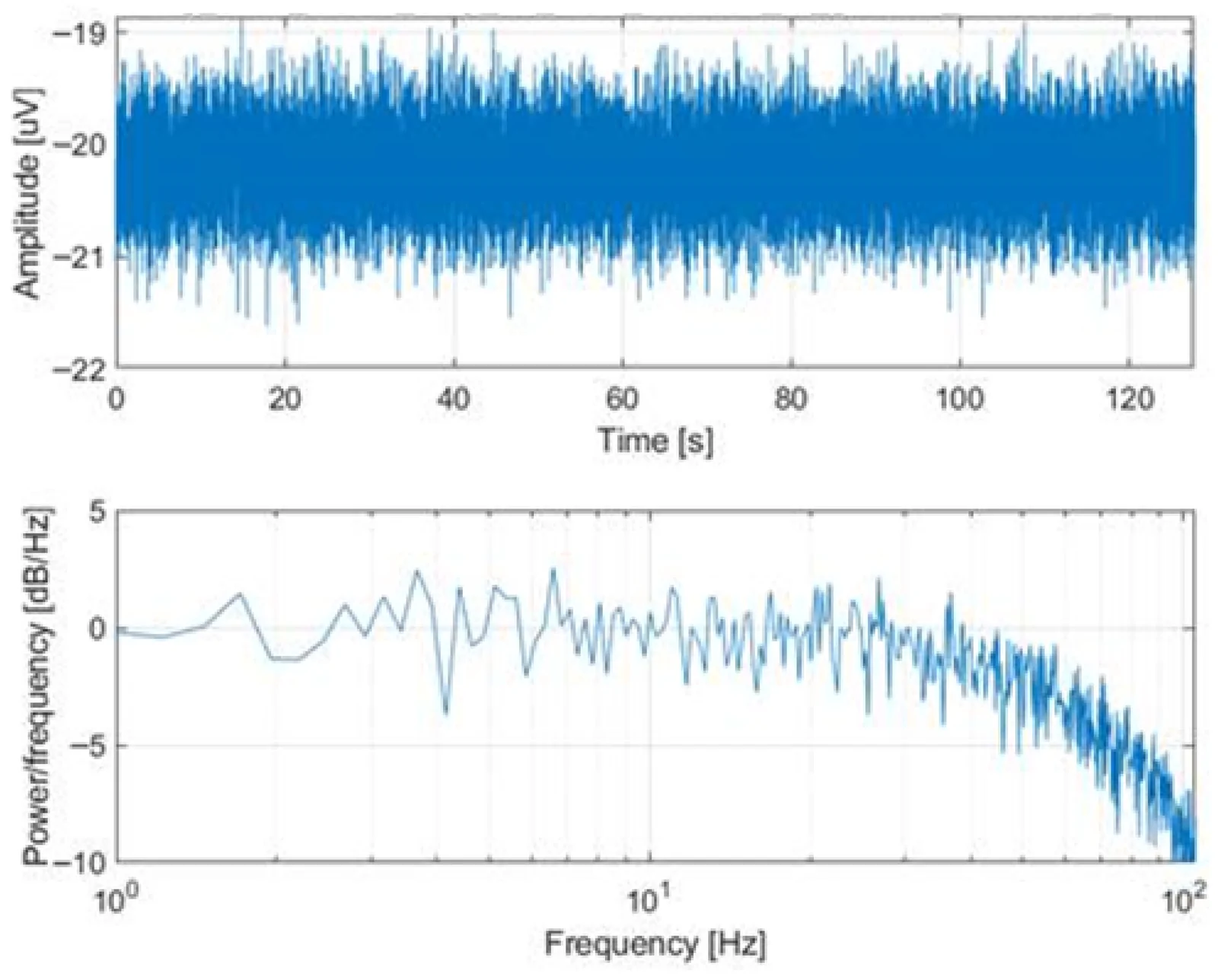
Characterization of EEG noise.

**Figure 9:**
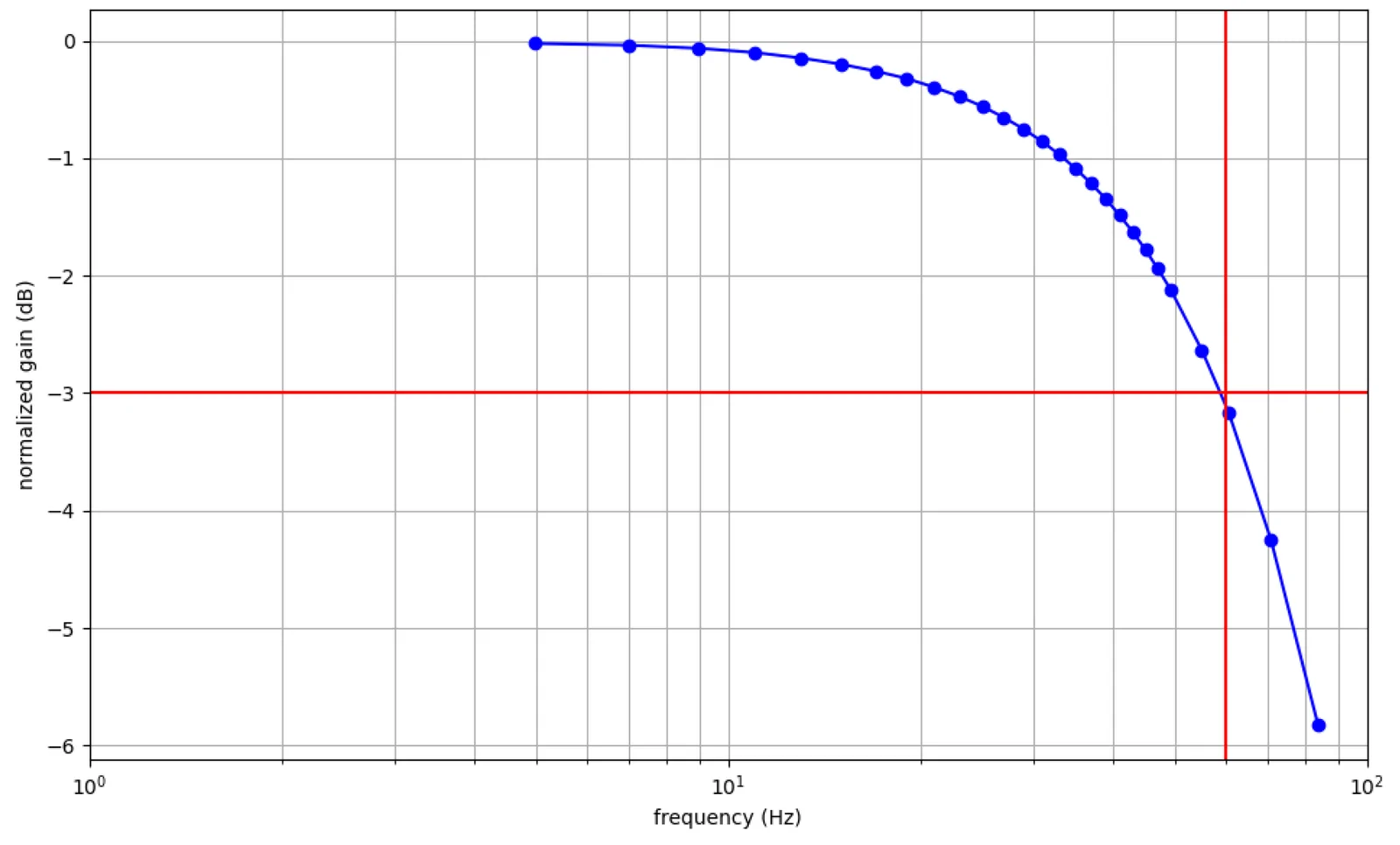
The 60 Hz bandwidth of the EEG system (The blue line represents the Bode diagram and red lines show the −3 dB bandwidth limit).

In order to characterize the noise floor of the EEG system, an acquisition is made with all EEG input short-grounded including the reference electrode (REF) and driven right leg circuit (DRL) special input. During this measurement, no fNIRS activation is enabled. Figure 8 shows the equivalent input signal in those conditions. The input-referred noise floor of 0.34 μV is the standard deviation of this signal. Using a Fast Fourier Transform (FFT), the normalized power spectral density of the total input noise is computed. The bandwidth of the system is approximately 60 Hz. This bandwidth has also been checked through a test procedure of channel stimulation with a consecutive single-tone pure sinus wave from an arbitrary wave generator (AWG). The normalized Bode chart of the system gain is shown in Figure 9. The normalization is conducted using the theoretical 13.8 dB gain (gain max: Rf = 2000 kΩ) of the AFE1299. This Bode chart confirms the 60 Hz bandwidth of the system.

#### 3.1.2. fNIRS Performances

In order to characterize the fNIRS system, a preliminary acquisition is made on an epoxy resin phantom of known optical properties at 740 nm ($\mu_{s}^{\prime }$ = 10 cm^−1^ and $\mu_{a}$ = 0.1 cm^−1^) representative of biological tissues. It is used to define the expected ratio between $P_{\mathrm{LED}}$ and $P_{\mathrm{PhD}}$ at the most restrictive long distances and also characterize the fNIRS system response for these targeted ratios. We measured the ratio $P_{\mathrm{PhD}}/P_{\mathrm{LED}}$ equal to $2\times 10^{6}$ at a mean distance of 3.5 cm. It means that for an incident power of 8.77 mW and 19.8 mW at $\lambda_{1}=$ 740 nm and $\lambda_{2}=$ 855 nm, respectively (with a current of 100 mA, see Figure 6), the system should be able to detect at least 4.4 nW and 9.9 nW at this distance.

**Figure 10:**
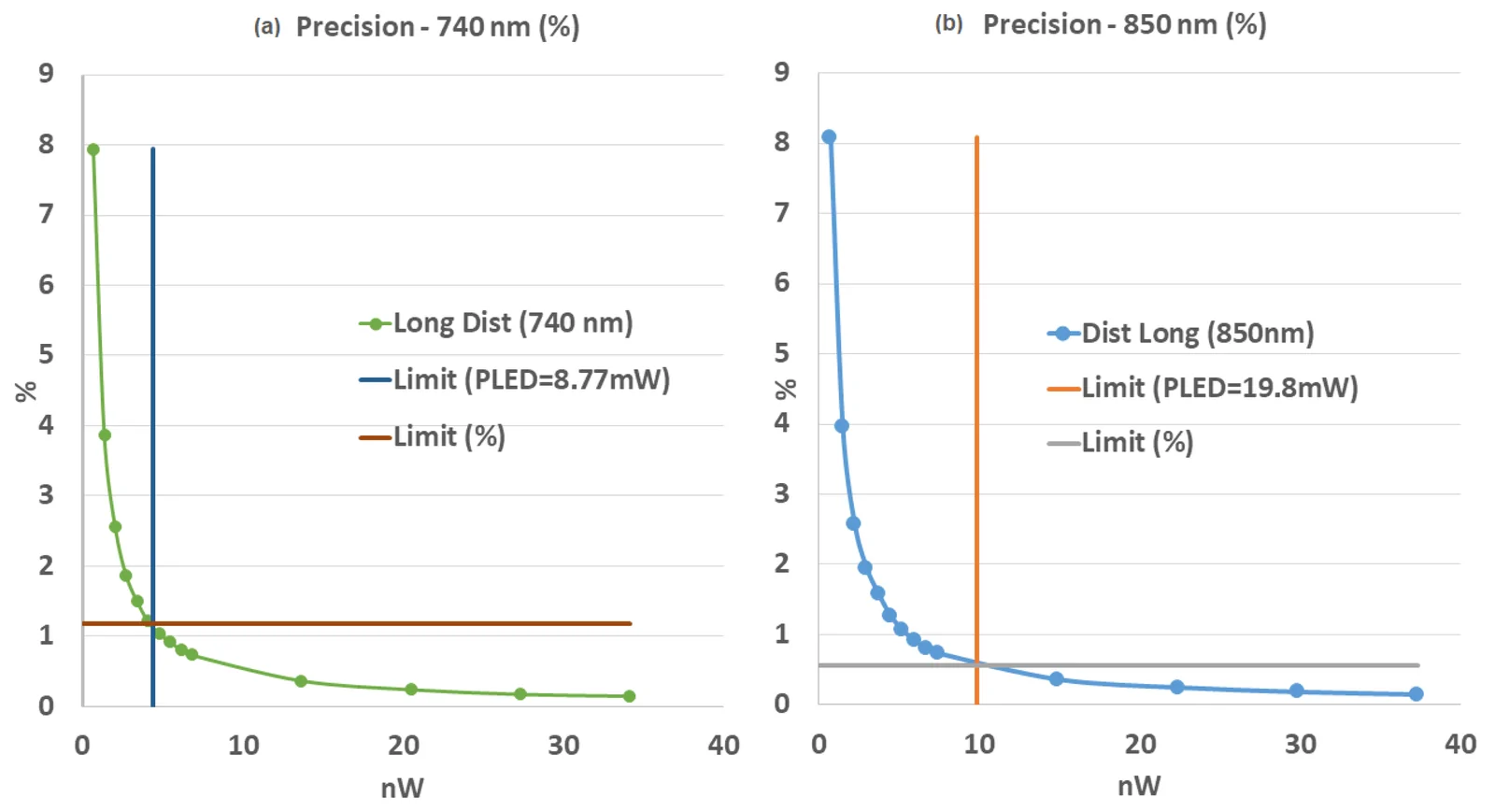
Precision of the detected fNIRS signal on long distances at (**a**) 740 nm and (**b**) 850 nm. Limit (PLED) represents the maximum received light power for each wavelength and Limit(%) shows the system precision for the maximum received light power.

We use the light measurement described in Section 2.4.2 with the Thorlabs current ramp to define the system precision as the ratio (in percentage) of the standard deviation of the detected signal to its mean value (corrected by the instrument offset) at each current value. Thus, we obtain the precision versus the detected power ($P_{\mathrm{PhD}}$) in Figure 10 for the two photodiodes used for long-distance measurements. A precision of less than 1.2% is thus obtained for both wavelengths, meaning the capacity of the system to detect such low power at long distance. During these experiments, no EEG signals were acquired.

### 3.2. Crosstalk

In order to quantify the electrical crosstalk between both EEG/fNIRS modalities, a measurement has been made using only one active bimodal sensor. The issue is primarily related to the fNIRS modality, which can modify the EEG electrical measurement by electromagnetic disturbance and induced currents.

**Figure 11:**
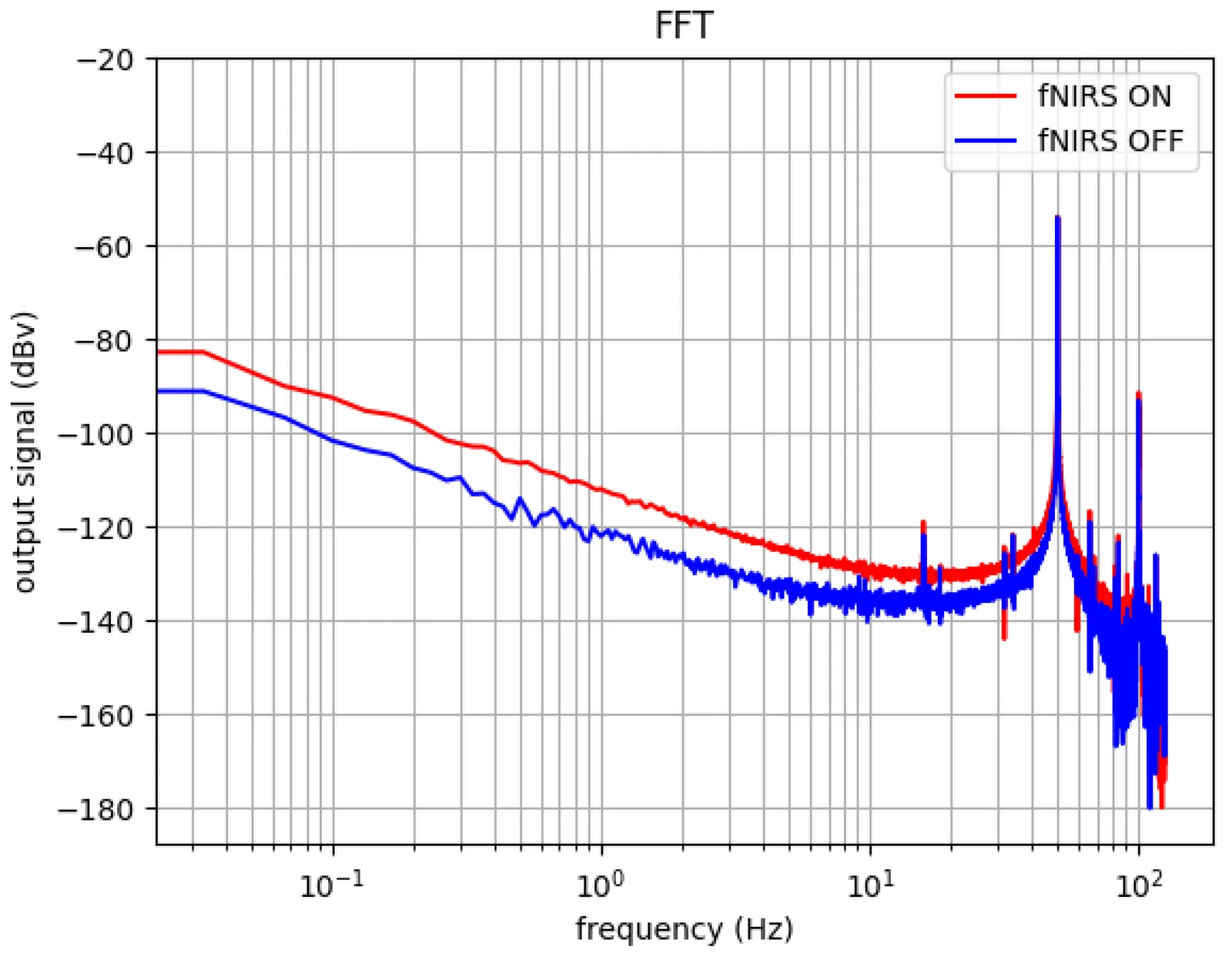
EEG spectrum with and without fNIRS activated.

The sensor is applied on a poor conductor surface with the REF and DRL. First, only the EEG is activated. In a second step, the fNIRS is activated. Figure 11 shows the FFT of the output signal. No filtering has been applied; thus, one can see the ambient 50 Hz spur, which will be notch filtered during signal processing. The noise floor is increased to 10 dB when the fNIRS modality is activated. This noise floor is still acceptable compared to the expected EEG signal strength. No spectral spurious is added, since the fNIRS data rate is 250 Hz, which is out of our band of interest.

## 4. Results

The Optrode system has been used in a study performed in collaboration with the Hospital team from Grenoble (lab HP2) Protocol number: 38RC22.0111, ID-RCB n: 2022-A00627-36. In total, 30 subjects participated in this study that contains, among other things, a calibration session and a cognitive task experiment. The system is used with five Optrode nodes located at forehead spatial positions AF7, AF3, AF8, AF4, FPz in the standard international 10–20 system. We also added a commercial electrode for electro-oculography (EOG) to deal with ocular artefacts and another commercial electrode in Pz as a benchmark to our Optrode system.

In the calibration session, we asked the subject to keep their eyes open for 180 s, keep their eyes closed for the same duration and then perform eye movements and blinks. This calibration session is intended to check if alpha waves are enhanced when the visual input is suppressed.

The protocol used to manipulate mental workload is an N-Back protocol with two conditions N=0 vs. N=2 [27]. In the 0-back condition, a letter $L_{n}$ is a target if it matches the first letter of the block $L_{1}$. In the 2-back condition, a letter $L_{n}$ is a target if it matches the letter $L_{n-2}$ from the same block.

Dedicated software has been developed for stimulus presentation and to log output (timing, keyboard response, …). During the letter presentation (500 ms), a white square is also present so that a photodiode directly placed on the screen captures the visual stimulus onset. This photodiode signal is digitized by one channel of the ADS1299. This trick allows precisely aligning an experimental paradigm with EEG/fNIRS data. A response pad allows the user to specify if the presented letter is a target or not, which in turn makes it possible to control the accuracy and the response time for each letter.

We will next demonstrate some EEG/fNIRS data collected during this study to show real data obtained with our sensor.

### 4.1. EEG/Calibration Session

**Figure 12:**
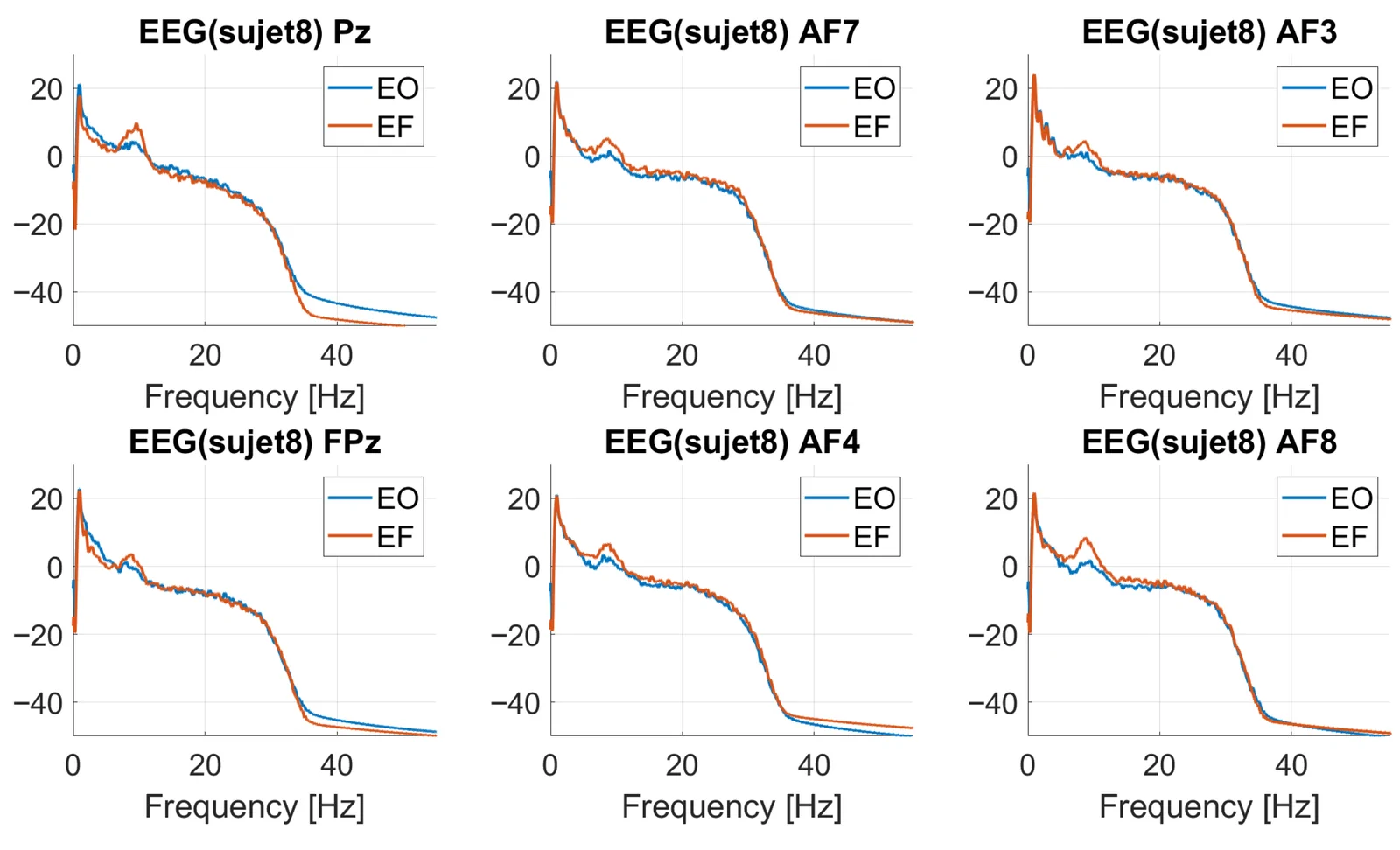
Eyes-open (blue) vs. eyes-closed (red) power spectra for one subject (S8).

A first validation protocol consists of verifying the ability of our system to detect the alpha spectral activity when a subject’s eyes are closed. The two-step protocol consists of a 60 s open-eyed stare followed by a 60 s closed-eyes stare. For each eyes-open/eyes-closed condition, a spectral analysis was performed with the pwelch method (window size of length 5000 ms). Prior to the spectral analysis, a Chebychev type-2 band-pass filter [0.5,40] Hz has been applied onto the EEG data. We superimpose for all sensor locations the power spectra in Figure 12.

As expected, the alpha power increased at every frontal/parietal electrode location in the closed-eyes condition with an individual alpha frequency (IAF) around 8.7 Hz for this subject [28].

**Figure 13:**
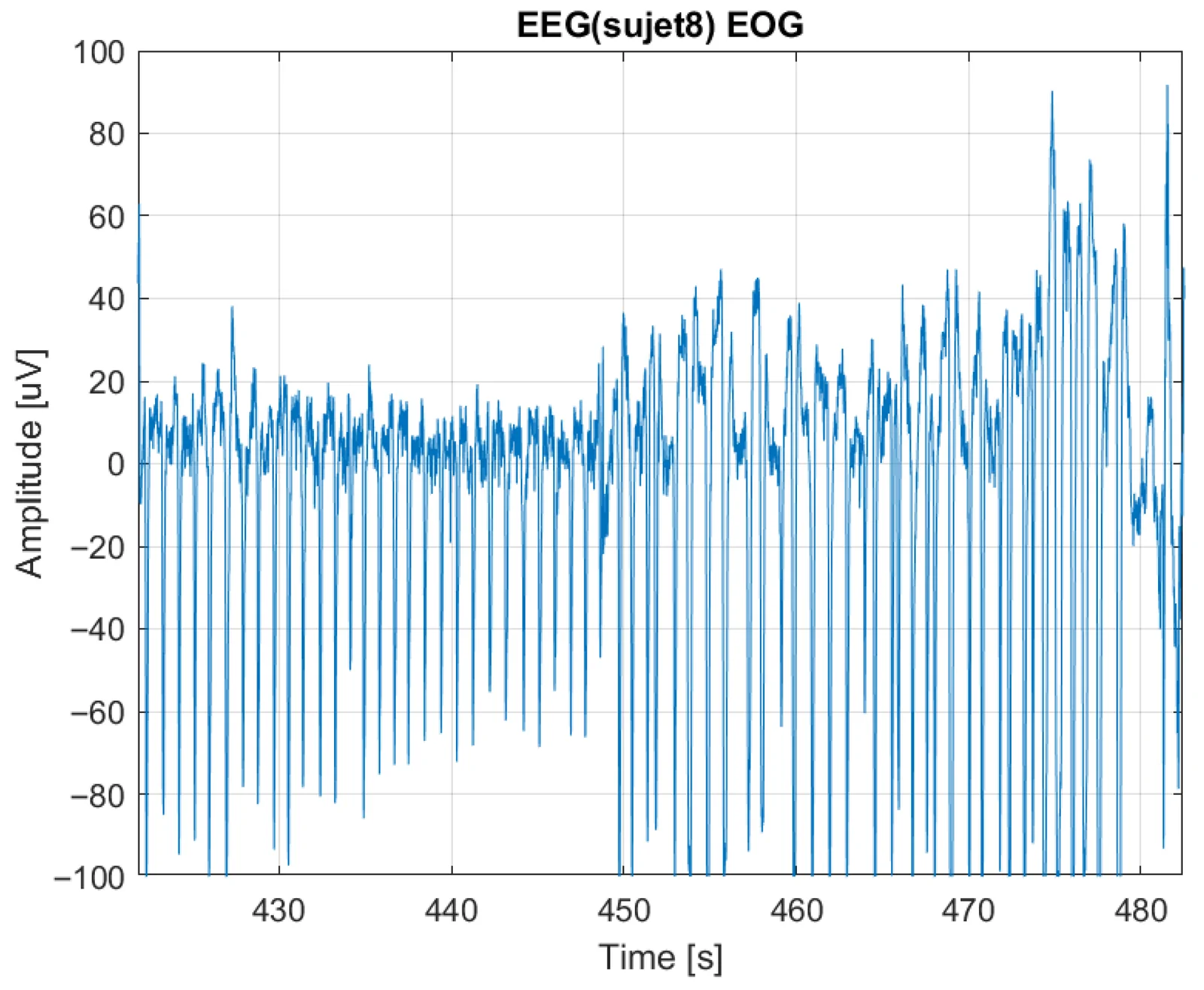
EOG electrode during eye movements.

As another validation, we also investigated the eye movements as recorded from EOG electrodes in Figure 13. We observe larger amplitude signals with repetitive patterns because of the eye movements. This signal can be used to identify and correct EEG artefacts.

### 4.2. fNIRS Quality Check

**Figure 14:**
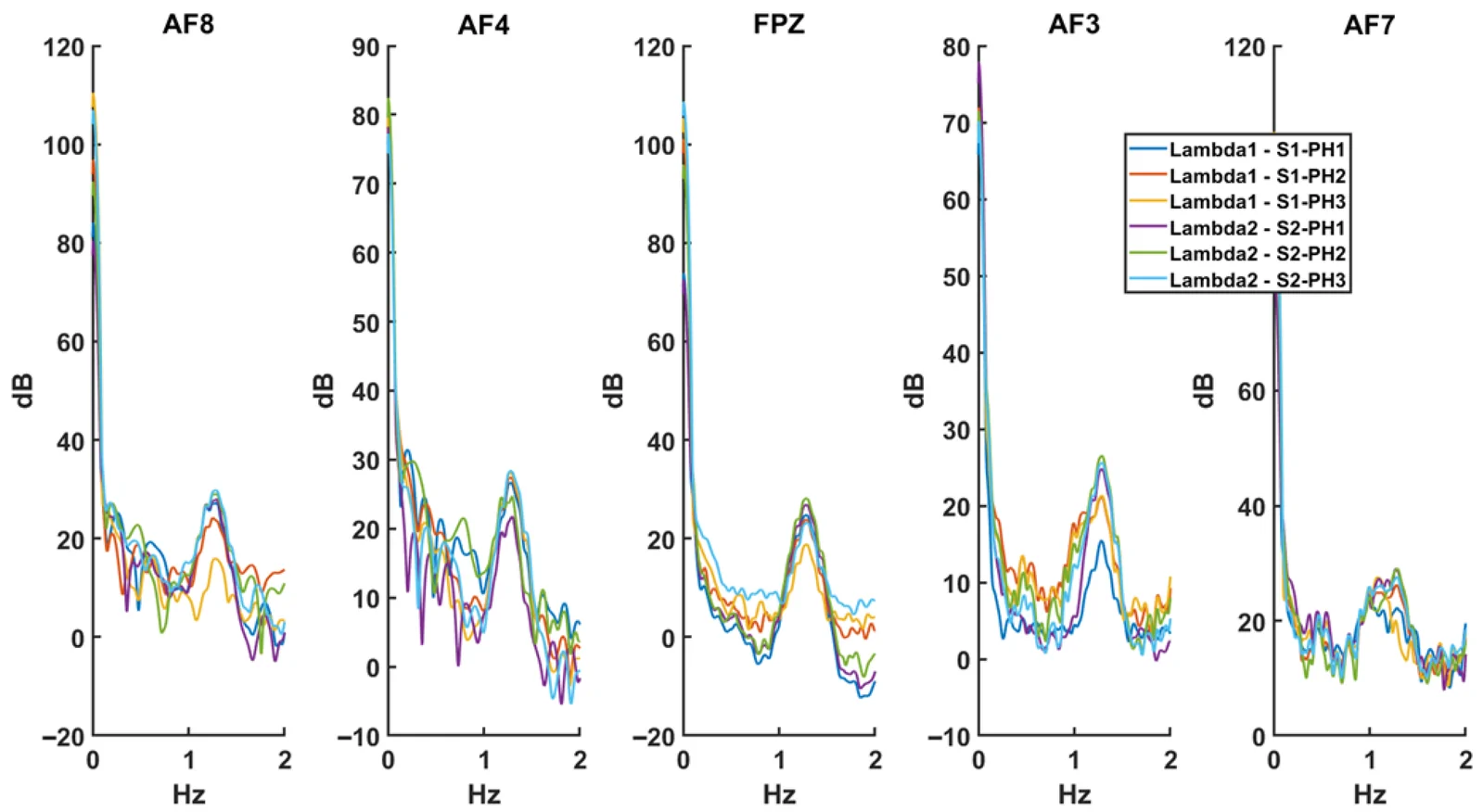
Results on 5 Optrode nodes placed on the forehead.

This check is about determining the quality of fNIRS signals—beyond motion artifacts—to eliminate irrelevant signals. The method implemented is based on work by [29], according to the method of Perdue et al. [30]. The FFT of the raw complete signal (raw data) was calculated (see Figure 14). A local fit on the heart rate-centered interval was achieved, and an amplitude of 12 dB compared to the background was considered sufficient to have a good SNR. Below this, the SNR may be limited (∼10 dB) or insufficient.

It is a question of validating an adequate SNR over long distances but also eliminating signals with a strong signal but little physiological content, which can happen on the short-distance (SD) channel, or a too weak and not significant short-distance signal—bad contact, presence of hair, etc.

### 4.3. fNIRS Processing

The modified Beer–Lambert (MBL) method is based on the photon propagation model [23] which defines the backscattered light intensity *I* at time *t*, distance *d*, and wavelength λ according to

(3) $$I\left(d,\lambda ,t\right)=I_{0}\exp\left[-d\cdot DPF\left(\lambda \right)\cdot \mu_{a}\left(\lambda ,t\right)+G\right]$$

where $I_{0}$ is the emitted light intensity.

The reflectance is defined as

(4) $$R\left(d,\lambda ,t\right)=\frac{I(d,\lambda ,t)}{I_{0}}$$

In this expression, $\mu_{a}$ is the absorption coefficient of the medium. DPF denotes the differential pathlength factor that depends both on the absorption and the scattering coefficient of the medium. It is taken equal to DPF=6 in this work. The *G* term is a constant depending on the diffusion properties of the medium and the geometry of the probe.

Introducing the attenuation signal as

(5) A(d,λ,t)=−lnR(d,λ,t)

we immediately see that the temporal variations of the attenuation ΔA(λ,t) between two instants $t_{1}$ and $t_{2}$ are linearly related to the fluctuations in $\mu_{a}$

(6) $$\Delta A\left(d,\lambda ,t_{1},t_{2}\right)=d\cdot DPF\cdot \Delta \mu_{a}\left(d,\lambda ,t_{1},t_{2}\right)$$

In pratice, ΔA is computed according to our corrected signal (Equation (2)) at distance *d*:

(7) $$\Delta A\left(\lambda ,d,t_{1},t_{2}\right)=\ln\left[{\tilde{V}}_{PhD}\left(d,\lambda ,t_{2}\right)\right]-\ln\left[{\tilde{V}}_{PhD}\left(d,\lambda ,t_{1}\right)\right]$$

Assuming further that the absorption change in the tissue results from the contributions of deoxy- (Hb) and oxy-hemoglobin (HbO_2_), we can further write the following:

(8) $$\Delta \mu_{a}\left(\lambda ,t\right)=\epsilon_{\mathrm{Hb}}\left(\lambda \right)\Delta c_{\mathrm{Hb}}\left(t\right)+\epsilon_{{\mathrm{HbO}}_{2}}\left(\lambda \right)\Delta c_{{\mathrm{HbO}}_{2}}\left(t\right)$$

Here, each chromophore is characterized by its concentration $c_{i}\left(t\right)$ [M] and by its extinction coefficient $\varepsilon_{i}\left(\lambda \right)$ [cm^−1^/M].

According to omlc.org, the molar extinction coefficients expressed in [cm^−1^/M] are $\epsilon_{{\mathrm{HbO}}_{2}}\left(\lambda_{1}\right)=446,\epsilon_{{\mathrm{HbO}}_{2}}\left(\lambda_{2}\right)=1058,\epsilon_{\mathrm{Hb}}\left(\lambda_{1}\right)=1115.88,\epsilon_{\mathrm{Hb}}\left(\lambda_{2}\right)=691.32$.

Assuming that only deoxy- (Hb) and oxy-hemoglobin (Hb0_2_) change, two wavelengths are required to estimate $\Delta c_{\mathrm{Hb}}$ and $\Delta c_{{\mathrm{HbO}}_{2}}$ at each time point.

The most commonly used method to avoid the extra-cerebral compartment contamination is the so-called short source–detector-distance-corrected MBL approach (SD-MBL) introduced by [31] and developed by many authors since then.

These approaches involve a short source–detector (SD) distance that is only sensitive to the surface layer and a long distance SD that is sensitive to the surface and deep layers [25]. The short distance is used as a regressor to remove the contribution of the surface to the measured signal at long distance according to the optical attenuation $A_{cerebral}=A_{PhD_{2}}-\beta A_{PhD_{1}}$ with β estimated according to different schemes (LMMSE, LMS, RMS). A similar correction is performed for the long-distance signal on $PhD_{3}$. Following preliminary work [32] concerned with the localization of the short distance, recent work has shown that extra-cerebral hemodynamic changes are spatially heterogeneous. These works therefore argue for a consideration of local short distances (by a hardware design allowing the measurement of these short distances at several places on the head) rather than a global numerical approach (using principal or independent component analysis methods, for example) [21,29]. Thus, our Optrode module includes the colocated acquisition of at least one short distance and one long distance.

### 4.4. N-Back: Protocol

#### 4.4.1. EEG/N-Back: Event-Related Potentials

We can analyze the brain activity elicited by the visual presentation of each letter. EEG data are epoched on the photodiode onset in the interval [−200,600] ms. ERP is obtained by averaging evoked responses over target trials in both conditions. Prior to epoching, a band-pass filter [2,30] Hz has been applied onto the EEG data. Trials with exceeding variances are rejected from the averaging process to suppress ocular and myogenic artefacts.

**Figure 15:**
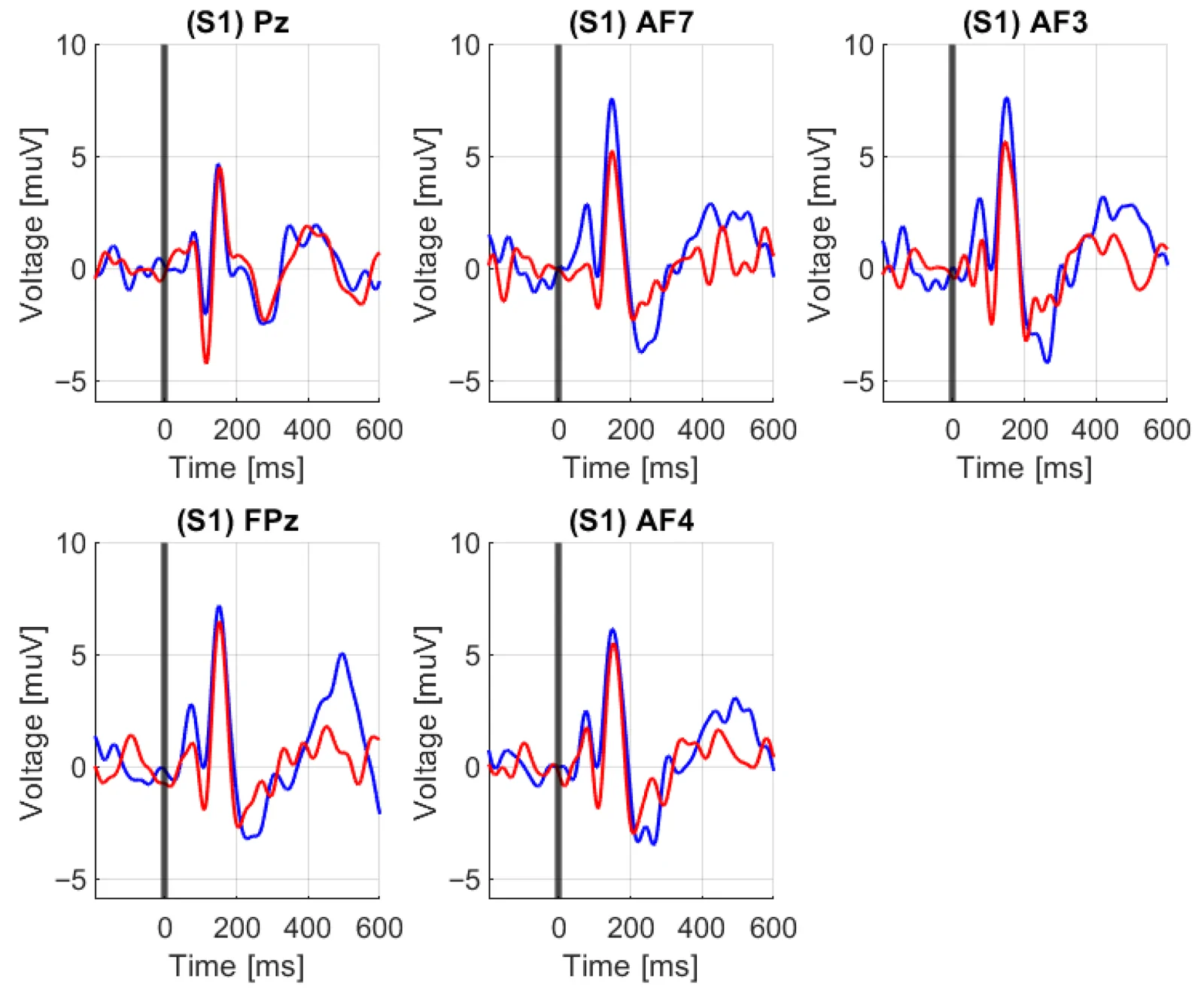
Target ERP. (Blue) 0-back, (Red) 2-back.

Figure 15 displays such ERP captured on a frontal site. The early component at t∼100 ms is reduced in amplitude when the mental workload is high [27]. The P300 component is hardly visible at this location.

#### 4.4.2. fNIRS Analysis

**Figure 16:**
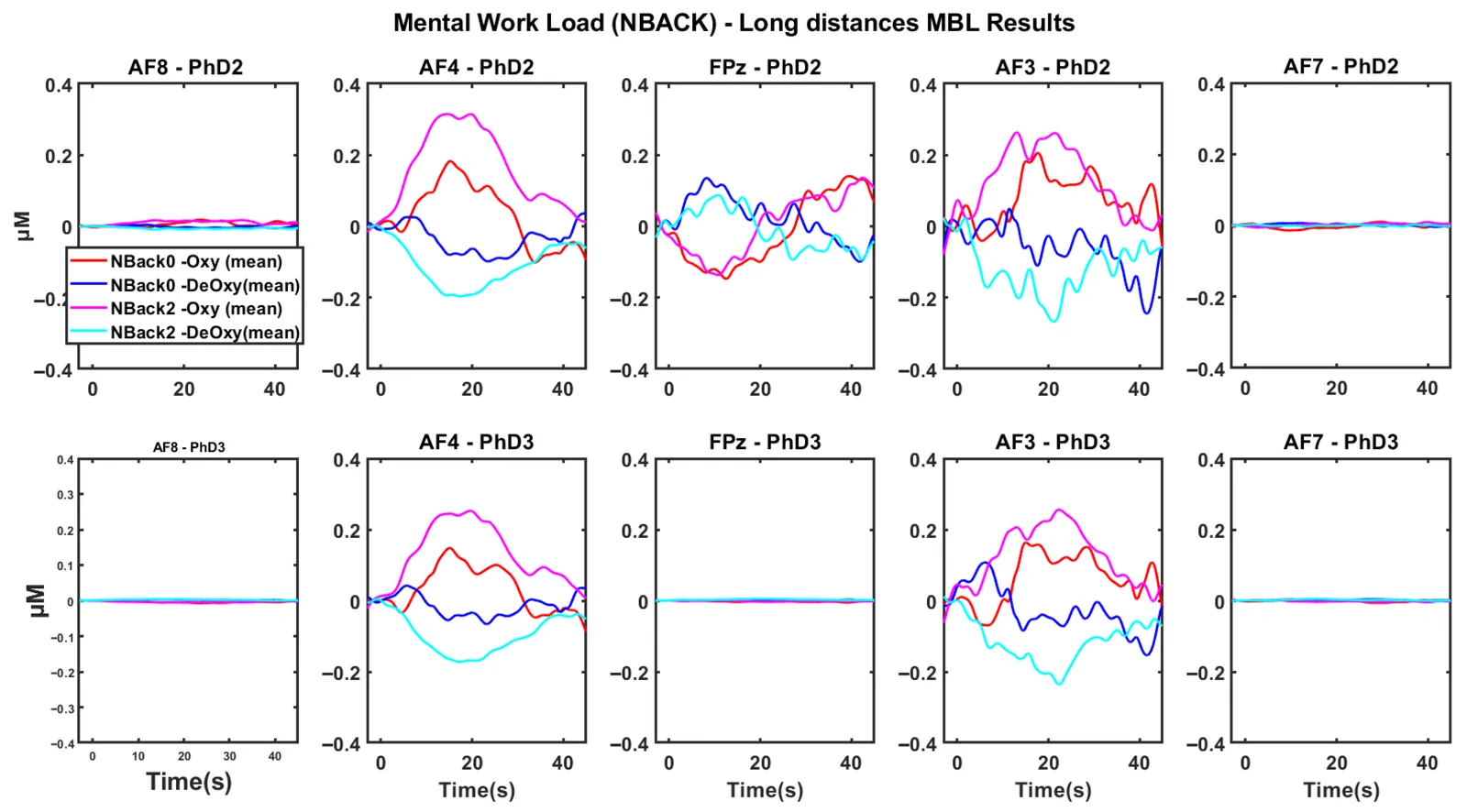
Results on 5 Optrode sensors placed in AF7, AF3, AFZ, AF4, and AF8 (from right to left. First line PhD2, second line PhD3. Block over 45 s and baseline 3 s).

We figure a result in Figure 16. fNIRS data are preprocessed with a band-pass filtering [0.01, 0.3] Hz. Motion artifacts are corrected using Tukey’s biweight robust mean method [33].

The short-channel MBL analysis is then performed, and oxy- and deoxy-hemoglobin concentrations variations are averaged on N-Back blocks conditions. The variation is calculated with reference to a baseline taken upstream of the block for a few seconds, which is typically 3 s.

We observe clear hemoglobin activation on two specific zones, AF3 and AF4. Note that on AF7 and AF8, the signals are relevant but with a very low activation (compared with the same scale). The activation is characterized by an augmentation of the oxy-hemoglobin concentration (red or magenta) during the task and a decrease afterwards. The deoxy-hemoglobin, on the contrary, decreases during the task (blue or cyan) and then comes back to its initial level. The 0-Back task induces a lower response (red and blue to be compared to magenta and cyan, respectively) than the 2-Back. Moreover, the 2-Back task implies a faster mobilization of the hemoglobin resources than the 0-Back.

## 5. Discussion

While the ability of fNIRS to study cognitive functions no longer needs to be demonstrated, it is clearly identified as sensitive to physiological noise. Some cognitive functions may generate changes in systemic variables; these lead to hemodynamic or oxygenational changes in the brain and extra-cerebral compartments that are not related to neuronal activity. A detailed analysis of these contributions may be found in [21].

We have shown in the previous section the ability of our Optrode system to process an N-Back protocol for workload estimation; we determined the electrical brain activity based on the EEG event-related potential (ERP) approach and the related hemodynamic activation based on the modified Beer–Lambert approach with the correction that the short distance (SD-MBL) and both long distances could be used to monitor it.

We believe the geometry of our Optrode sensor offers the possibility to go further: unlike the SD-MBL analysis which relies on the temporal variation of the signals, the SRS—spatially resolved spectroscopy—analysis relies on the spatial variation of the signals measured between the two long distances at each time step.

Based on the Matcher model of photon propagation [34], $\mu_{a}$ is obtained according to the following:

(9) $$\mu_{a}≃\frac{1}{3\mu_{s}^{\prime }}{\left[\frac{\partial A}{\partial d}-\frac{2}{d}\right]}^{2},\;\frac{\partial A}{\partial d}≃\frac{A\left(PhD_{3}\right)-A\left(PhD_{2}\right)}{d_{3}-d_{2}}$$

With the known scattering $\mu_{s}^{\prime }$ hypothesis, $\mu_{a}$ is derived from (9), and absolute concentrations are derived from (8).

Here, $d_{k}$ denotes the source to the *k*-th photodiode detector, and *d* is obtained as the mean of ($d_{2},d_{3}$).

The calculation of this gradient can be tricky due to the weak signals involved at long distances; the calibration steps should be carefully defined so that such low signals can be compared. If well done, the SRS approach leads to the estimation of the absolute values of oxy- and deoxy-hemoglobin, where the MBL approach estimates their variations only. Moreover, the spatial (SRS) and temporal (SD-MBL) analysis of the signals can help in the interpretation of the systemic and neurological activity. Indeed, a systemic response present in both extra-cerebral and cerebral compartments would not be identified by the SD-modified Beer–Lambert approach (due to regression), whereas it would be identified by the SRS approach.

Even if the combination of the two approaches is beyond the scope of this paper, we believe our Optrode sensor can help to better interpret complex brain responses when the objective is to leave the laboratory. Moreover, the 250 Hz data rate of the short distance make it possible to exploit it as a plethysmography signal with the estimation of physiological parameters such as heart rate or variation in heart rate. These parameters, together with the neuroparameters estimated by SD-BLM or SRS, can allow addressing complex contexts related to the real environment.

## 6. Conclusions

In this article, a complete EEG/fNIRS system has been described. It compares well with other previous works both in terms of EEG performance and fNIRS capability, as seen in Table 3. It allows the acquisition of electrical and hemodynamic brain activity data through its bimodal geometry. This geometry allows taking advantage of the good temporal resolution of the EEG and the high spatial resolution of the fNIRS. The electrical galvanic isolation of both modalities allows the simultaneous acquisition at 250 Hz with crosstalk reduction. The innovative fNIRS modality consists of a short distance and two long distances that allow for SD-MBL and SRS data processing. This device is modular by design: it allows placing the Optrode sensors anywhere on the forehead to allow a measurement of frontal and prefrontal activity. The sensors are light, comfortable and can be worn by subjects for one hour without any problems. Tests were conducted with this system out of the laboratory on a cohort of more than 30 subjects. The results of this study have demonstrated encouraging performances of our system in combining EEG and fNIRS modalities to improve mental state classification even with a limited covering of the brain region (forehead). Those results will be published in a second paper dedicated to our signal processing methodology.

## Figures and Tables

**Table 1:** Sensitivity response normalized with respect to the PhD1 response $c_{ij}$ for calibration for an optical power of 500 nW/cm^2^. Single asterisks indicate nominal values for short distances, and double asterisks indicate nominal values for long distances.

$R_{f}\;\left[k\Omega \right]$	$c_{21}$ PhD2/PhD1 @740 nm	$c_{31}$ PhD3/PhD1 @740 nm	$c_{22}$ PhD2/PhD1 @850 nm	$c_{32}$ PhD3/PhD1 @850 nm
10	106%	111%	110%	117%
25 *	106%	112%	110%	117%
50	106%	112%	110%	118%
100	106%	112%	110%	118%
500	106%	112%	109%	118%
1000	106%	112%	109%	118%
2000 **	107%	113%	111%	119%

**Table 2:** Quantum yield $\eta_{i2}$ for each photodiode for an optical power of 500 nW/cm^2^. Single asterisks indicate nominal values for short distances, and double asterisks indicate nominal values for long distances.

$R_{f}\;\left[k\Omega \right]$	$\eta_{12}$ PhD1	$\eta_{22}$ PhD2	$\eta_{32}$ PhD3
10	120%	116%	114%
25 *	120%	117%	115%
50	121%	117%	115%
100	121%	117%	115%
500	120%	116%	114%
1000	120%	117%	114%
2000 **	120%	116%	114%

**Table 3:** System performances comparison with other EEG/fNIRS systems.

	[19]	[26]	[35]	[17]	This Work
EEG noise floor (μVrms)	0.89	0.14	0.29	0.44	0.345
EEG sampling rate (Hz)	16k	250	250	2k	250
fNIRS sampling rate (Hz)	100	5	8	10	250
EEG resolution (bits)	24	24	24	12	24
fNIRS resolution (bits)	24	16	24	12	22
short and long distance fNIRs	0 + 1	0 + 1	0 + 1	0 + 2	1 + 2
Dry EEG electrode	yes	yes	no	yes	yes
Co-located EEG/fNIRS	yes	no	no	no	yes
Crosstalk suppression	yes	yes	yes	no	yes
Sensor dimension (mm^2^)	78.54	na	716 × 60	na	50 × 20

## Data Availability

The data presented in this study are available on request from the corresponding authors. The data are not publicly available due to privacy or ethical restrictions.
